# Restorative potential of laser-synthesized silver nanoparticles with *Salvia officinalis* for periodontal disease treatment: an in vitro study

**DOI:** 10.3762/bjnano.17.55

**Published:** 2026-06-15

**Authors:** Jelena Filipović Tričković, Sanja Živković, Bojana Ilić, Miloš Tošić, Jelena Marinković, Ana Valenta Šobot, Miloš Momčilović

**Affiliations:** 1 Department of Physical chemistry, „VINČA” Institute of Nuclear Sciences - National Institute of thе Republic of Serbia, University of Belgrade, Mike Petrovića Alasa 12-14, 11351 Belgrade, Republic of Serbia

**Keywords:** cell proliferation, periodontal disease, pulsed laser ablation in liquid (PLAL), *Salvia officinalis*, silver nanoparticles (AgNPs)

## Abstract

Periodontal disease is one of the most prevalent human pathologies initiated by microbial dysbiosis and the inflammatory reaction that leads to gingival fibroblasts apoptosis, connective tissue damage, and atrophy, resulting in tooth loss. Beyond local damage, systemic associations highlight the urgent need for innovative therapies. Herein, we present an eco-friendly synthesis of silver nanoparticles (AgNPs) by picosecond pulsed laser ablation in liquid (PLAL), using *Salvia officinalis* aqueous extract (sage extract) as both medium and stabilizer. AgNPs were synthesized at two laser pulse energies (2 and 6 mJ), in sage extract and deionized water, and characterized by dynamic light scattering, transmission electron microscopy, and inductively coupled plasma optical emission spectroscopy. Synthesis at 6 mJ in sage extract (_Sage_AgNPs_6mJ_) yielded well-dispersed, spherical nanoparticles (7.98 ± 2.9 nm) at a concentration of 21.6 ± 1.75 mg·L^−1^. _Sage_AgNPs_6mJ_ exhibited potent antibacterial activity against *Aggregatibacter actinomycetemcomitans*, *Fusobacterium nucleatum*, and *Porphyromonas gingivalis* (MIC = 5.4 mg·L^−1^), and showed equal or superior antibacterial activity compared to 0.2% chlorhexidine. Importantly, at MIC concentration, _Sage_AgNPs_6mJ_ were non-cytotoxic to primary human gingival fibroblasts and enhanced their proliferation by approximately 50% after 24 h. This was shown by increased Ki-67 proliferation index, increased type-I and total collagen production up to 11% after 24 h and significantly reduced IL-6 by 23% after 24 h, which returned to the baseline levels after 48 h. These findings define a clear therapeutic window, where the same concentration that eradicates bacteria also promotes gingival cell regeneration and reduces inflammation. This dual antibacterial and regenerative action demonstrates the high potential of sage-assisted PLAL-synthesized AgNPs as a safe, sustainable strategy for periodontal disease treatment.

## Introduction

Synthesis of silver nanoparticles (AgNPs) is a promising nanotechnology with numerous biomedical applications, especially in the field of tissue restoration and regeneration [1]. The most commonly used methods in AgNP synthesis are (i) chemical methods, which employ silver nitrate as a precursor and different reducing agents as a stabilizer, (ii) biological methods, which use natural precursors like bacteria, enzymes, and plant extracts, and (iii) physical methods based on arc discharge, sputtering, physical vapor deposition, or laser ablation [2]. While chemical synthesis offers size control, it typically involves toxic reducing agents, raising biocompatibility concerns [3]. AgNP synthesis using plant extracts has gained attention due to environmental friendliness, but it lacks precise control over size and dispersity of AgNPs. Laser synthesis, specifically pulsed laser ablation in liquid (PLAL), involves laser ablation of a metal target immersed in a liquid, upon which a laser plasma is produced and nanoparticles are generated [4]. To the best of our knowledge, although PLAL is traditionally associated with lower yields compared to conventional chemical synthesis [5–6], recent advances in laser systems have significantly improved its scalability. Importantly, the absence of chemical reagents and purification steps reduces downstream processing costs, making PLAL a promising and economically feasible approach for the production of high-purity AgNPs in biomedical applications. PLAL addresses the limitations of both biosynthesis and chemical synthesis by offering a reproducible, contaminant-free method with high reproducibility and size control. Combining laser ablation of a metal target with natural compounds is a unique concept that provides not only a clean, environmentally friendly method that avoids the use of hazardous chemicals; the obtained nanoparticles could also retain the properties of both nanoparticles and plant extract, thus achieving synergistic effects.

*Salvia officinalis* L. (sage) is a medicinal herb commonly used in dentistry with anti-inflammatory, antigenotoxic, and anti-plaque properties [7]. While several studies reported the green synthesis of AgNPs by the chemical reduction of AgNO_3_ using sage extract [8–12], to the best of our knowledge, the picosecond laser ablation of a silver metal target using aqueous sage extract as a stabilizer has not been reported. In the work conducted by Déné and co-workers [8], antifungal properties of sage-synthesized AgNPs were reported; but neither antibacterial effects nor effects on the eukaryotic cells were evaluated, and the synthesis relied on a chemical approach. In contrast, Mostafavi and colleagues [10], investigated the wound-healing potential of combined AgNP and sage extract treatment in an in vivo mouse skin model, indicating possible synergistic effects between silver and sage. Nevertheless, the antibacterial activity was not evaluated, and sage was not utilized in the nanoparticle synthesis itself. Importantly, none of these studies reported the exact silver concentrations used in treatments, which limits the assessment of their safety and potential clinical applicability. We have previously demonstrated that sage leaf extract obtained by aqueous extraction and lyophilization exhibits potent antioxidant, antigenotoxic, and immunomodulatory activities with potential applications in preventing or treating inflammatory conditions [13]. Additionally, we have shown that AgNPs synthesized by PLAL using citrate solution possess antibacterial activity and can modulate cell viability and oxidative stress in a size- and concentration-dependent manner [14]. Based on these findings, we hypothesize that AgNPs produced by PLAL in an aqueous sage extract environment could maintain the bioactive properties of both AgNPs and sage extract, leading to enhanced biological effects.

Periodontal disease is considered to be one of the most common diseases in humans with prevalence estimated to be approximately 42% to 62% of the world population, among which 7.8% to 23.6% are severe forms, according to the National Health and Nutrition Examination Surveys data [15–16]. Its origin stands on microbial dysbiosis induced by the maturation of dental plaque, which, in susceptible hosts, leads to polymorphonuclear and mononuclear cell infiltration, initiating gingival inflammation followed by excessive production of pro-inflammatory cytokines. Progressively, connective tissue damage occurs, beginning with fibroblast alteration and secretion of metalloproteinases, which degrade collagen and other components of the extracellular matrix [17–18]. Eventually, fibroblast apoptosis and inhibition of collagen production ensue, causing overall gingival atrophy and irreversible damage to the periodontal ligament. Current strategies for the prevention and the treatment of periodontitis are oriented towards improved dental hygiene, scaling and root planning, but also restrictive or regenerative surgical approaches [19–20]. Optimal treatment should be oriented towards the elimination of pathogenic microbes and reduction of inflammation and gingival atrophy, but also toward collagen production.

This study aimed to evaluate the biological properties of AgNPs synthesized by PLAL, where the varying parameters were different laser pulse energies (2 and 6 mJ) and distinct synthesis media, namely, (i) aqueous sage extract and (ii) deionized water. To the best of our knowledge, this study is the first to report the synthesis of AgNPs via PLAL using aqueous sage extract as a stabilizing medium. The objective was to investigate the influence of synthesis parameters on AgNP properties and to evaluate their biological effects, including antibacterial activity against the key periodontal pathogens *Porphyromonas gingivalis*, *Fusobacterium nucleatum*, and *Aggregatibacter actinomycetemcomitans*, and modulatory effects on primary human gingival fibroblast cell (GFC) viability, proliferation, collagen production, and inflammatory responses. This approach aims to explore the potential effects of PLAL-generated AgNPs in sage extract for improved periodontal treatment strategies.

## Materials and Methods

### Preparation and characterization of aqueous sage leaf extract

Aqueous sage leaf extract (sage) was prepared prior to nanoparticle synthesis, as previously described [13]. Commercially available *S. officinalis* leaves (Serial No. 23380723, Institute of Medicinal Plant Research “Dr Josif Pančić”, Belgrade, Serbia) were ground, infused with boiling water, and cooled to room temperature. The extract was filtered (5–13 μm, Lab Logistic Group), frozen at −20 °C overnight, and lyophilized under 400 Pa vacuum pressure. The lyophilizate was subsequently filtered (0.2 μm, Minisart^®^, Sartorius) and reconstituted in sterile water to a concentration of 100 mg·mL^−1^. After preparing the extract, the concentration of selected phenolic acids was analyzed.

### Analysis of selected phenolic acids in sage extract

Sage extract was dissolved in deionized water, homogenized, and used to determine the content of selected phenolic acids, that is, vanillic, caffeic, ferulic, and rosmarinic acids using high-performance chromatography (HPLC) with a diode array detector. Phenolic acids were selected according to literature data regarding biologically active sage constituents that possess reducing and stabilizing roles in AgNP formation [21–25]. A series of phenolic acids standard solutions (Merck, Darmstadt, Germany) in water in the concentration range of 5–500 μg·mL^−1^ was prepared. 5 μL of sample was injected and separation was performed on a Zorbax Eclipse XDB-C18 column (150 mm × 4.6 mm, 5 μm) at 35 °C. The components were eluted with a mobile phase based on a 0.1% aqueous solution of formic acid (A) and methanol (B), at a flow rate of 1.0 mL·min^−1^, in gradient mode: 0 min 10% B, 10 min 10% B, 20 min 20% B, 30 min 70% B, 40 min 100% B. The UV–vis signal was monitored in the range of 200–600 nm. To determine the content of phenolic acids, chromatograms were recorded at 220, 260, 290, and 325 nm. To determine the content of vanillic acid, the areas of the peaks from the chromatogram recorded at 260 nm were read, and for caffeic and rosmarinic acids the ones at 325 nm. Phenolic acid concentrations were determined using the external standard method. Chromatograms were processed in ChemStation software (Agilent Technologies).

### Laser synthesis of AgNPs

AgNP synthesis was performed by PLAL of a silver metal target (silver foil, 1 mm thickness, 99.99% purity, Alfa Aesar, USA) using a picosecond Nd:YAG EKSPLA SL 212/SH/FH laser system (EKSPLA, Lithuania) operating at the fundamental wavelength of 1064 nm with a 150 ps pulse duration and a repetition rate of 10 Hz. Two different pulse energies (2 and 6 mJ), corresponding to fluences of 0.04 and 0.12 J·cm^−2^ and a laser-irradiated area of approximately 0.05 cm^2^ on the target surface, were used with or without sage extract to evaluate the influence of laser energy on nanoparticle synthesis. The laser beam was focused through a lens having a focal length of 15.2 cm with the target placed at 16.2 cm from this lens, that is, under slightly defocused conditions. An automated *x*–*y* translation stage ensured continuous movement of the target during ablation so that fresh surface irradiation could be achieved. Four AgNPs samples were obtained, namely, _Sage_AgNPs_2mJ_ and _Sage_AgNPs_6mJ_, which were synthesized in the presence of sage extract (100 µL), as well as _dw_AgNPs_2mJ_ and _dw_AgNPs_6mJ_, which were prepared in 8 mL of deionized water (18 MΩ·cm) under the same laser conditions. After synthesis, characterization of the AgNP samples was performed.

### AgNP characterization

The total concentration of silver in obtained colloidal solutions was determined using inductively coupled plasma optical emission spectroscopy (ICP-OES, iCap7400 DUO, Thermo Fisher, USA) and expressed as mg·L^−1^ ± SD.

The hydrodynamic diameters (dH) of the obtained nanoparticles were analyzed by using the dynamic light scattering (DLS) technique using a Zetasizer NanoZS90 (Malvern Panalytical, UK) with a 633 nm He–Ne laser source and 90° detection optics. The measurements were performed in disposable polystyrene cuvettes (DTS0012, Malvern Panalytical, UK) at ambient temperature (25 ± 0.1 °C).

The selected AgNPs that displayed antimicrobial effects were further analyzed by transmission electron microscopy (TEM), to evaluate their size, shape, and dispersity. An FEI Talos F200X microscope, operating at 200 keV with an X-FEG source and point-to-point resolution below 0.24 nm, was used to analyze nanoparticles after periods of two days and one month after synthesis. The photomicrographs were captured in the conventional mode and recorded on the CCD camera with a resolution of 4096 × 4096 pixels. The size distribution was measured manually, measuring at least 1000 nanoparticles using the image capture software (SemAfore, Version 5.21, Finland). Following the AgNP characterization, antibacterial testing, as well as the assessment of their effects on human GFCs was performed.

### Experimental design and treatments

Antimicrobial testing was performed to determine the minimal inhibitory concentrations (MICs) of all obtained AgNPs. Upon antimicrobial assessment, cytotoxicity evaluation was done to investigate the range of non-cytotoxic AgNP concentrations by testing concentrations of 0.5 × MIC, 1 × MIC, and 2 × MIC. The proliferative capacity of selected, non-cytotoxic treatments was analyzed to estimate the restorative potential of selected AgNPs. Finally, following the identification of AgNPs that possess antibacterial properties and stimulate cell proliferation, we evaluated their potential to impact collagen production (type-I and total collagen production) and inflammation reduction by monitoring levels of interleukins IL-1β and IL-6.

### Antimicrobial testing

Following AgNP synthesis and characterization, the antibacterial potential was evaluated in microdilution assays against *Aggregatibacter actinomycetemcomitans* (ATCC 29552), *Porphyromonas gingivalis* (ATCC 33277), and *Fusobacterium nucleatum* (ATCC 25586) in accordance to ISO 20776-1. The strains were cultured in Shaedler broth (Thermo Fisher Scientific, Waltham, MA, USA) supplemented with vitamin K and hemin at 37 °C for 72 h in an anaerobic chamber.

MICs as well as minimal bactericidal concentrations (MBCs) were determined according to the method previously described in [26]. Inoculum containing 2 × 10^4^ CFU per well (10^5^ CFU·mL^−1^) of bacteria was added to each well after the serial dilution of the NPs. After the 24 h incubation, resazurin was used as a growth indicator, and it was introduced at a final concentration per well of 0.0675 mg·mL^−1^. After 3 h of incubation with resazurin, wells with no color changes (with no visible bacterial growth) and the lowest concentration were determined as those with the MICs of NPs. Additionally, MBCs were determined by plating from the wells with no noticeable growth onto Brucella blood agar.

### Cytotoxicity testing (XTT assay)

The cytotoxic effect of AgNPs was determined on human primary GFCs provided by courtesy of the School of Dental Medicine, Department of Biology and Human Genetics, University of Belgrade. All volunteers read and signed the informed consent, and the study was conducted in agreement with the Declaration of Helsinki and approved by the Ethics Committee of the Vinča Institute of Nuclear Sciences, Serbia (approval no. 116-17-2/2020-000).

The cytotoxicity assessment was performed by applying the 2,3-bis-(2-methoxy-4-nitro-5-sulfophenyl)-2*H*-tetrazolium-5-carboxanilide (XTT) assay according to the standard procedures [27]. Upon seeding in 24-well plates at a density of 0.05 × 10^6^ and reaching the subconfluent level, the cells were treated with concentrations of 0.5 × MIC, 1 × MIC, and 2 × MIC for 24 and 48 h. Absorbance was measured at 470 nm on a microplate reader (Sunrise, Tecan Group Ltd., Switzerland). The results are presented as the percentage of viable cells compared to the untreated control (100% of viable cells).

### Cell proliferation assay

Ki-67 immunofluorescent staining was performed to analyze the effects of AgNPs on the proliferation rate of GFCs. The cells were seeded on polylysine-coated slides (Merck, Germany) and treated with selected AgNPs for 24 and 48 h. Following treatment, the cells were fixed with 4% formaldehyde solution, permeabilized with 0.25% Triton-X (Merck, Germany), and blocked with 1% bovine serum albumin (BSA, Merck, Germany). The cells were hybridized overnight at 4 °C with directly labeled anti-Ki-67 antibody (SolA15, FITC, eBioscience™, Termo Fisher Scientific, USA). The slides were stained with DAPI-Vectashield solution (Vector Laboratories, United Kingdom) and analyzed using a Zeiss-Axioimager A1 microscope and the ISIS imaging software package (MetaSystems, Altlussheim, Germany). The results are presented as the proliferation index (PI), that is, the ratio between the number of immunoreactive (Ki-67^+^) cells and the total number of cells. At least 1000 cells per treatment were analyzed.

### Assessment of collagen production

The collagen production rate was monitored by immunofluorescent staining of type-I collagen and colorimetric detection of the total collagen content (TCC).

Type-I collagen immunofluorescent staining was performed on the GFCs seeded on polylysin-coated slides, as described above. After the treatment, fixation, and permeabilization, the cells were incubated overnight at 4 °C with type-I collagen polyclonal antibody (PA1-26204, Invitrogen, Thermo Fisher Scientific, USA), washed and incubated with goat anti-rabbit IgG (H+L) cross-adsorbed secondary antibody, Cyanine3 (Cy3, A10520, Invitrogen, Thermo Fisher Scientific, USA). Type-I collagen was monitored qualitatively using a Zeiss-Axioimager A1 microscope and the ISIS imaging software package.

Quantitative analysis of TCC after treatments was assessed using the Total Collagen Assay Kit (Abcam, Cambridge, United Kingdom) according to the manufacturer’s instructions. Briefly, after the treatments, cells were collected and lysed by freeze-thawing, and the protein concentration for each sample was determined as previously described [28]. Optical densities were detected by a microplate reader at 560 nm (Sunrise, Tecan). The collagen standard was used to construct the calibration curve, and the values were normalized per milligram of proteins in the samples. Results were presented as percentage of TCC compared to the control.

### Analysis of pro-inflammatory cytokine levels

The effects of AgNPs on inflammation were determined by measuring the levels of interleukin 1β (IL-1β) and interleukin 6 (IL-6) in GFCs after inflammation induction with 10 mg·mL^−1^ lipopolysaccharides from *Escherichia coli* (EcLPS, Scientific Laboratory Supplies Ltd, United Kingdom) [29]. Interleukins were determined in cell culture media using the commercial LEGEND MAX™ Human IL-1β and IL-6 ELISA Kits (BioLegend, Inc., San Diego, CA, USA) according to the manufacturer’s instructions. The optical densities were measured at 450 nm on a microplate reader (Sunrise, Tecan). The results are presented as pg·mL^−1^ of IL-1β and IL-6.

### Statistical analysis

For all performed assays, two independent experiments were performed, each in three replicas. The results are presented as mean value ± standard error of mean (SEM). Statistical analysis was performed using One-way ANOVA statistical test from SPSS 10 statistical package for Windows (IBM, Armonk, NY, USA). *P* values < 0.05 were accepted as the level of significance.

## Results

### Determination of selected phenolic acids

The analysis of selected phenolic acids in the aqueous sage extract in this study showed that among the four phenolic acids analyzed, rosmarinic acid was the most abundant (41.1 ± 0.8 mg·g^−1^) and caffeic acid was found at a concentration of 410 ± 20 µg·g^−1^, while ferulic and vanillic acids were below the detection limit.

### AgNP synthesis and characterization

Four types of AgNPs were synthesized by silver target ablation in deionized water or sage extract using two different laser pulse energies (2 and 6 mJ) (Table 1). The reported values represent the total silver concentration in the colloidal solution and do not directly reflect nanoparticle number concentration due to differences in particle size distribution. Although the applied pulse energies are relatively low, the corresponding fluences are sufficient to induce efficient ablation due to the short pulse duration of the picosecond laser. Our results showed that higher laser pulse energy (6 mJ) and surrounding sage extract increase total silver concentration and reduce the hydrodynamic diameters (dH). The smallest nanoparticles with the highest concentration were produced by applying a laser energy of 6 mJ in the sage solution (_Sage_AgNPs_6mJ_).

**Table 1:** AgNP synthesis parameters, concentrations (ICP-OES), and hydrodynamic diameters (DLS).

AgNP type	Laser energy	Ag concentration (mg·L^−1^ ± SD)	Sage extract concentration (mg·L^−1^)	Hydrodynamic diameter, dH (nm ± SD)

_Sage_AgNPs_2mJ_	2 mJ	8.4 ± 1.5	800	89.6 ± 17.2
_Sage_AgNPs_6mJ_	6 mJ	21.6 ± 1.75	800	14.6 ± 3.1
_dw_AgNPs_2mJ_	2 mJ	3.5 ± 1.2	—	115.5 ± 21.4
_dw_AgNPs_6mJ_	6 mJ	15.2 ± 2.1	—	68.4 ± 16.2

**Figure 1:**
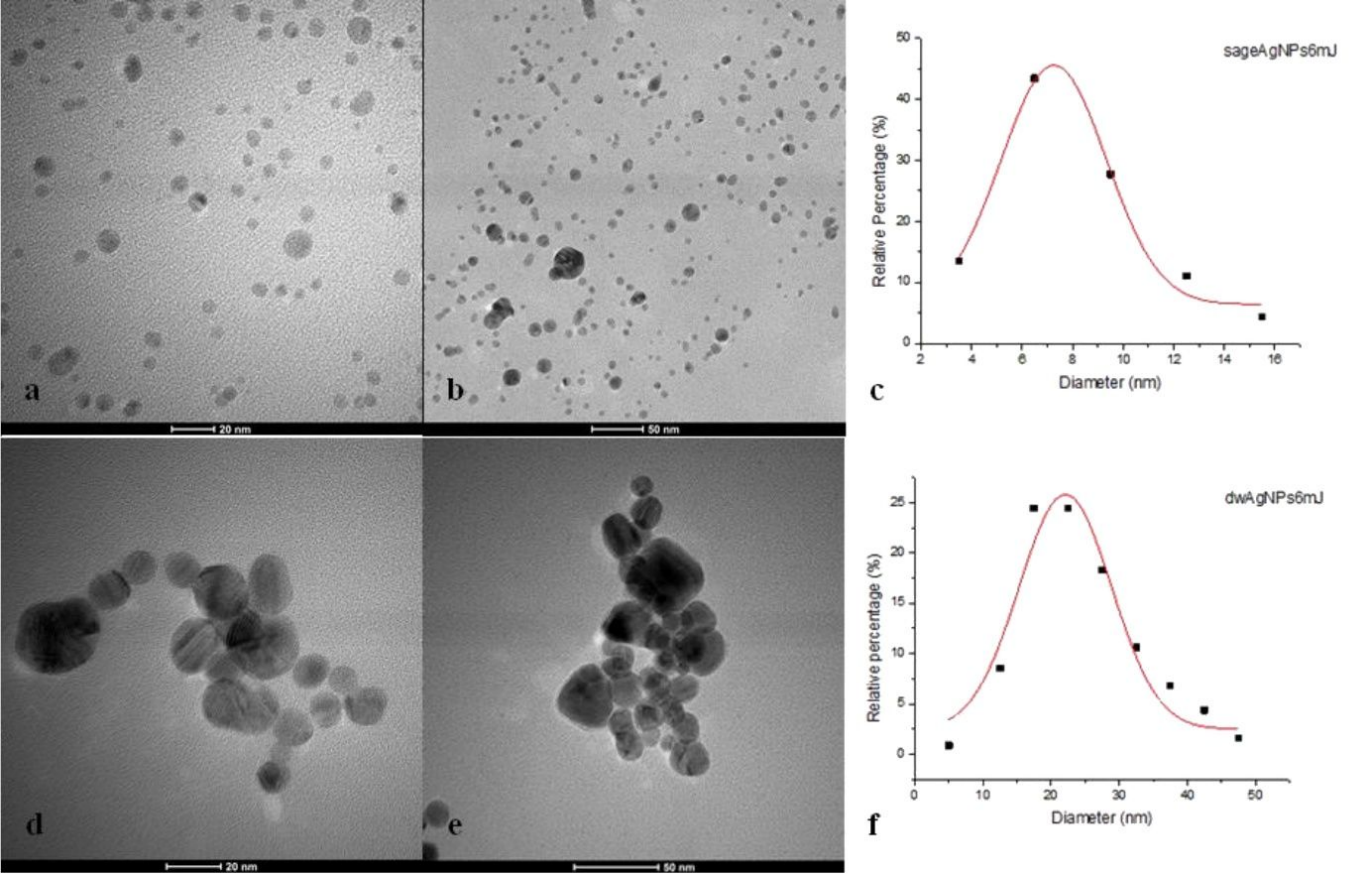
Photomicrographs of _Sage_AgNPs_6mJ_ and _dw_AgNPs_6mJ_ two days after synthesis (a and d, scale bar 20 nm), and one month after synthesis (b and e, scale bar 50 nm) and corresponding log–normal particle size distributions two days after synthesis (c and f) measured by TEM.

The two AgNP types with the higher concentration and the smaller particle size, with and without sage extract, that is _Sage_AgNPs_6mJ_ and _dw_AgNPs_6mJ_, respectively, underwent TEM analysis. _Sage_AgNPs_6mJ_ were well dispersed, spherical, and in the narrow size range from 2.1 to 16.3 nm, with an average particle size of 7.98 ± 2.90 nm. Minor agglomeration after one month was observed, suggesting high stability of the nanoparticles (Figure 1a,b). _dw_AgNPs_6mJ_ were spherical and pseudospherical in shape, with particle diameters ranging from 9.0 to 48.8 nm, with an average particle size of 24.59 ± 8.25 nm, with minor agglomeration that was more pronounced after one month (Figure 1d,e).

### Antibacterial testing

The primary step in exploring the AgNPs’ potential in periodontal disease management was the determination of their inhibitory potential against the most common periodontopathogens. AgNPs synthesized using the low laser energy of 2 mJ, did not display any antibacterial activity (Table 2).

**Table 2:** Antibacterial activity of AgNPs, sage extract, and 0.2% chlorhexidine against *Aggregatibacter actinomycetemcomitans*, *Fusobacterium nucleatum*, and *Porphyromonas gingivalis*.^a^

	*Aggregatibacter actinomycetemcomitans*		*Fusobacterium nucleatum*		*Porphyromonas gingivalis*	

	MIC (mg·L^−1^)	MBC (mg·L^−1^)	MIC (mg·L^−1^)	MBC (mg·L^−1^)	MIC (mg·L^−1^)	MBC (mg·L^−1^)

_Sage_AgNPs_2mJ_	n.d.	n.d.	n.d.	n.d.	n.d.	n.d.
_Sage_AgNPs_6mJ_	5.4	5.4	5.4	5.4	5.4	5.4
_dw_AgNPs_2mJ_	n.d.	n.d.	n.d.	n.d.	n.d.	n.d.
_dw_AgNPs_6mJ_	7.6	7.6	7.6	7.6	7.6	7.6
sage extract	25000	50000	12500	25000	12500	25000
0.2% chlorhexidine	6.62	6.62	6.62	6.62	3.31	3.31

^a^n.d. – not determined.

In contrast, both AgNP samples synthesized with the higher laser energy of 6 mJ displayed high inhibitory potential against all tested bacterial strains, and _Sage_AgNPs_6mJ_ showed the highest antibacterial potential. The potential was even higher than that of 0.2% chlorhexidine against *A. actinomycetemcomitans* and *F. nucleatum* (Table 2)*.* The antibacterial effect of pure aqueous sage extract was achieved at much higher concentrations compared to the sage concentration in _Sage_AgNPs_6mJ_; the calculated sage concentration in the _Sage_AgNPs_6mJ_ colloidal solution at 1 × MIC concentration was 200 mg·L^−1^.

### Viability assessment and proliferation potential

**Figure 2:**
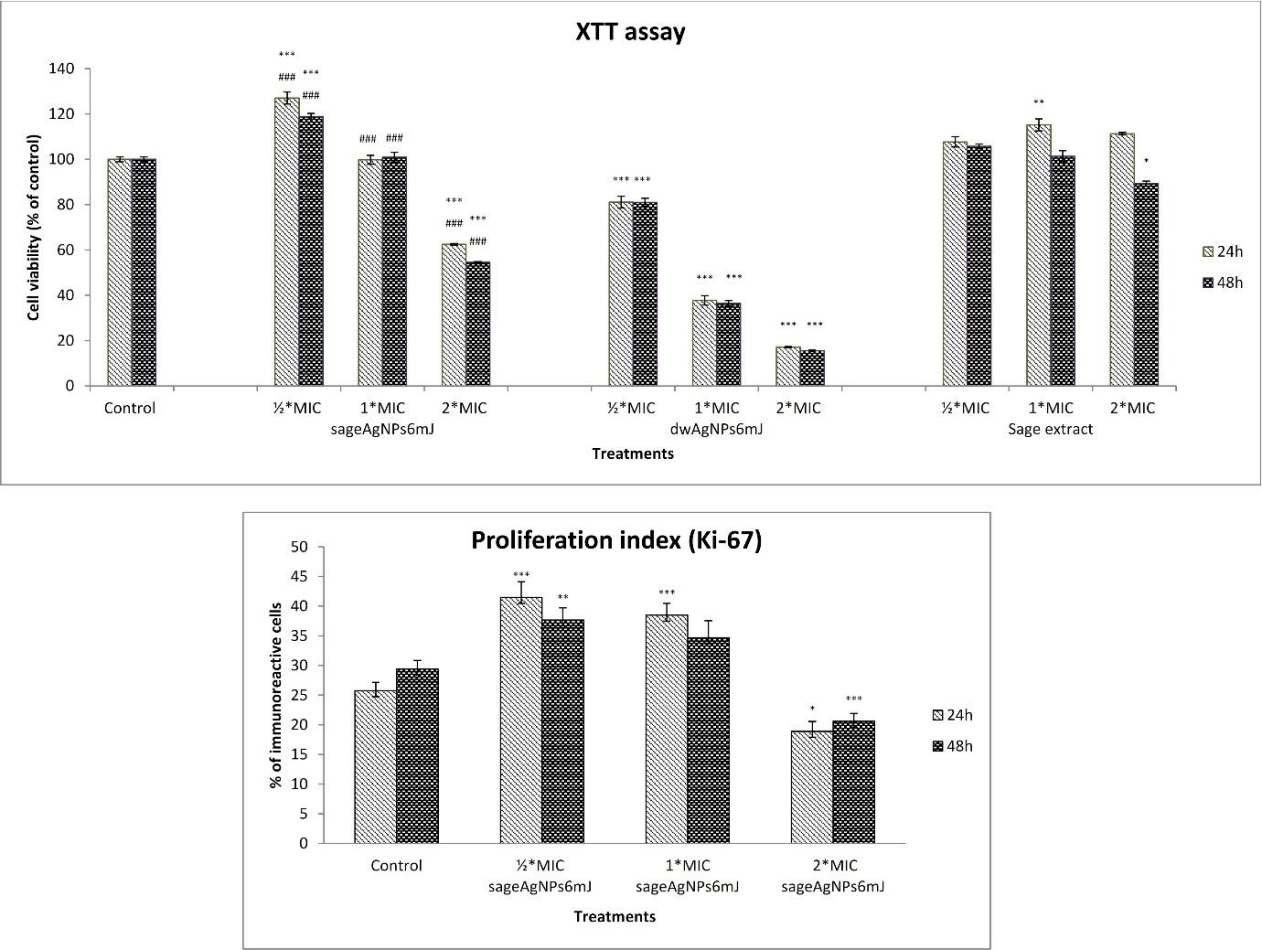
Cell viability after treatment with _Sage_AgNPs_6mJ, dw_AgNPs_6mJ_, and sage extract at concentrations corresponding to 0.5 × MIC, 1 × MIC, and 2 × MIC (a), and proliferation index after treatment with _Sage_AgNPs_6mJ_ (b) for 24 and 48 h. ^*^p < 0.05, ^**^p < 0.01, ^***^p < 0.001, treatments vs control; ^###^*p* < 0.001, _Sage_AgNPs_6mJ_ vs _dw_AgNPs_6mJ_.

**Figure 3:**
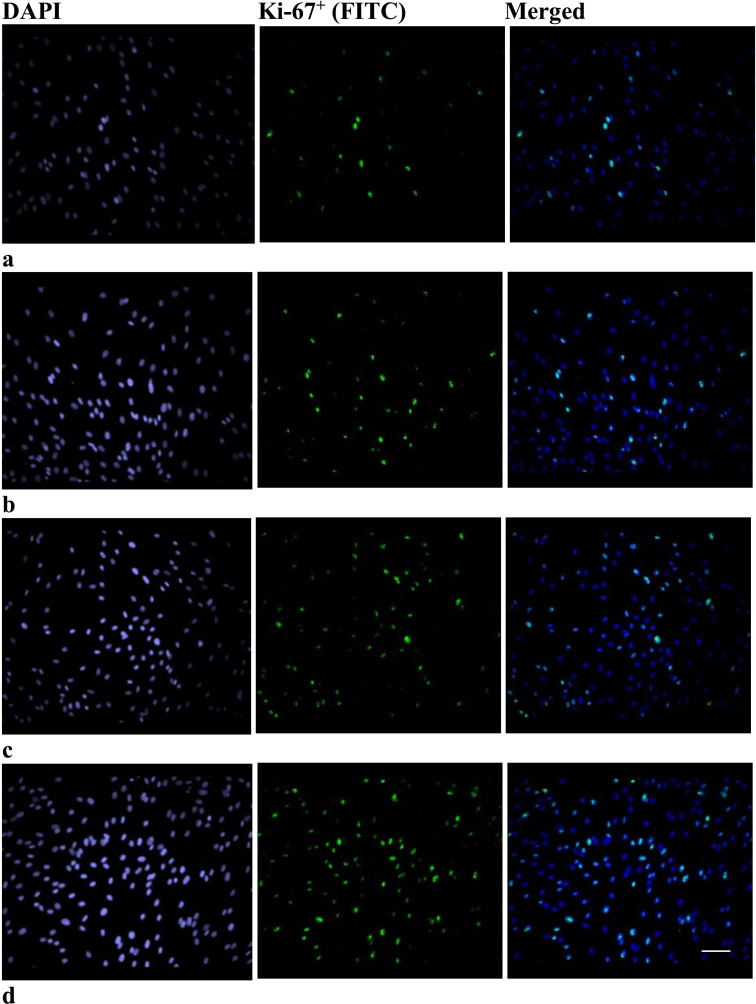
Ki-67 immunofluorescent staining of untreated GFCs after 24 h (a) and 48 h (c) and GFCs treated with _Sage_AgNPs_6mJ_ at 1 × MIC concentration for 24 h (b) and 48 h (d). The left panel shows DAPI-stained nuclei, the middle panel shows proliferative active Ki67^+^ cells (FITC labeled, green), and the right panel shows merged photomicrographs. Scale bar = 100 μm.

The viability of GFCs was evaluated after treatment with concentrations of 0.5 × MIC, 1 × MIC, and 2 × MIC of _Sage_AgNPs_6mJ_ and _dw_AgNPs_6mJ_ to identify the range of non-cytotoxic concentrations. The results of the XTT assay showed that _Sage_AgNPs_6mJ_ did not display cytotoxic effects after 24 and 48 h of treatment up to a concentration of 5.4 mg·L^−1^ (1 × MIC). In contrast, _dw_AgNPs_6mJ_ at the concentration of 1 × MIC (7.6 mg·L^−1^) significantly reduced the number of viable cells to 37.76% after 24 h of treatment (Figure 2a, *p* < 0.001). The reduction in cellular survival was greater after 48 h of treatment with _dw_AgNPs_6mJ_ (36.41%), suggesting a cytotoxic effect at 1 × MIC concentration after both exposure times. In contrast, pure sage extract led to a moderate increase in cell viability after 24 h of treatment, significant at the concentration that corresponds to 1 × MIC of _Sage_AgNPs_6mJ_ (*p* < 0.01), while 48 h of treatment induced mild concentration-dependent decrease in cell viability (Figure 2a). Considering the cytotoxic effects of the 1 × MIC concentration of _dw_AgNPs_6mJ_, the potential of _Sage_AgNPs_6mJ_ to accelerate the proliferation of GFCs was examined only for _Sage_AgNPs_6mJ_ by applying Ki-67 immunofluorescent staining. Concentrations of 0.5 × MIC and 1 × MIC of _Sage_AgNPs_6mJ_ increased GFC proliferation rate for both treatment times, highlighting their proliferative potential (Figure 2b and Figure 3). However, a higher concentration of 2 × MIC significantly reduced the proliferation index, supporting the XTT results and the cytotoxicity of higher concentrations.

### Assessment of collagen production

The identification of a “therapeutic window” in which _Sage_AgNPs_6mJ_ demonstrate antimicrobial efficacy against periodontal pathogens while also increasing GFC proliferation represents a critical advancement for potential clinical translation as it allows for effective bacterial elimination at concentrations that do not compromise host tissue integrity. Therefore, the monitoring of collagen production rates and interleukins levels was performed only for these sage-synthesized AgNPs at 1 × MIC concentration.

**Figure 4:**
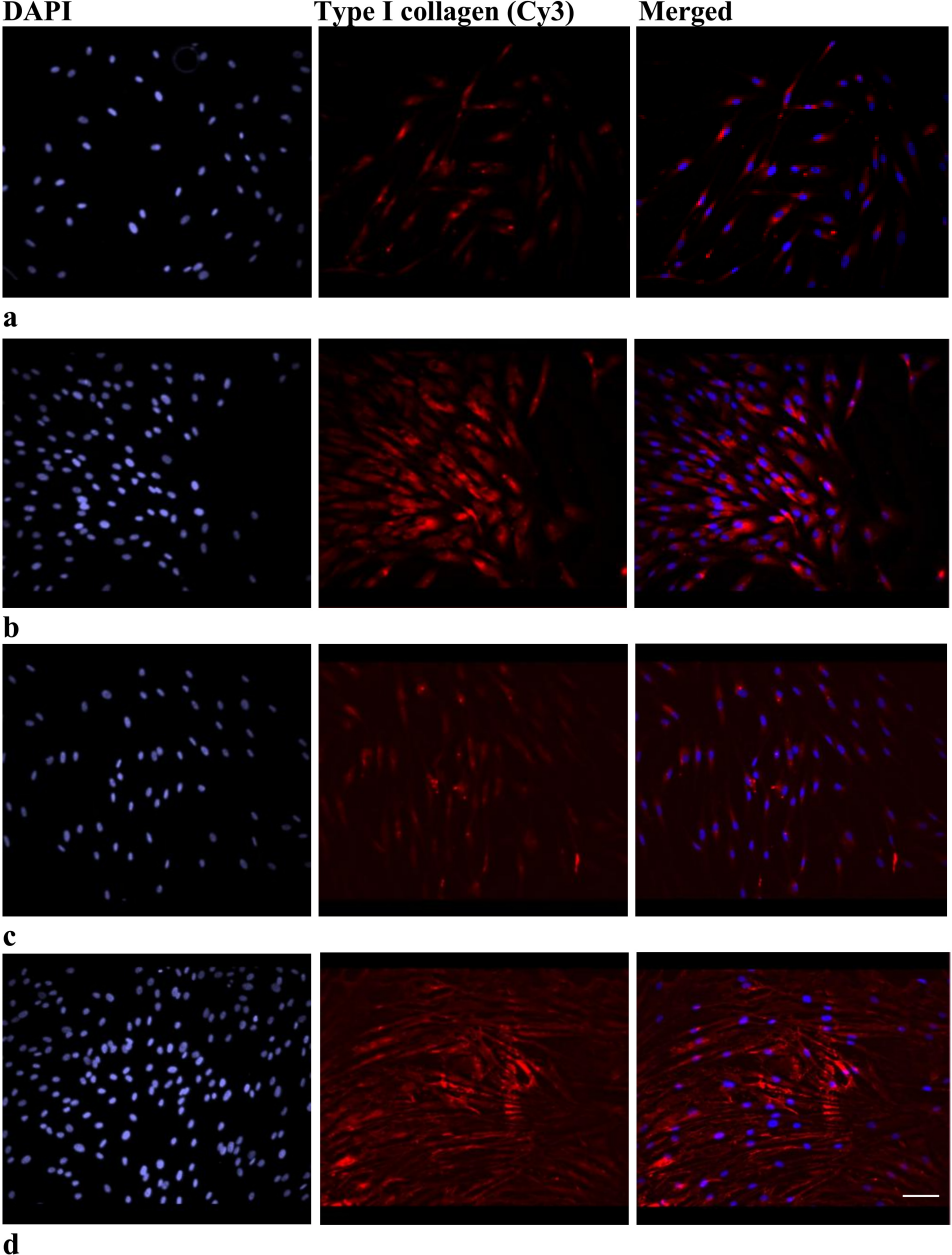
Photomicrographs of type-I collagen immunofluorescent staining of GFCs treated with _Sage_AgNPs_6mJ_ for 24 h (b) and 48 h (d), and of untreated cells (a, c). The left panel shows DAPI-stained nuclei, the middle panel shows type-I collagen (Cy3-labeled, red), and the right panel shows merged photomicrographs. Scale bar = 100 μm.

Immunofluorescent staining showed that after 24 h of treatment, type-I collagen production intensified compared to the untreated control (Figure 4b). 48 h of treatment induced a more pronounced difference in collagen production, causing not only an increase in intracellular type-I collagen, but also its extracellular secretion (Figure 4d). TCC analysis also showed a statistically significant increase in total collagen production rate by 11% after 24 h (from 100% in control, to 111.65% ± 0.94% in _Sage_AgNPs_6mJ_-treated cells) and 5% after 48 h (from 100% to 105.68% in _Sage_AgNPs_6mJ_-treated cells). The increase after 48 h of treatment is probably higher than the colorimetric measurement showed since extracellular collagen deposition of type-I collagen was observed through immunofluorescent staining (Figure 4d).

### Pro-inflammatory cytokine measurement

**Figure 5:**
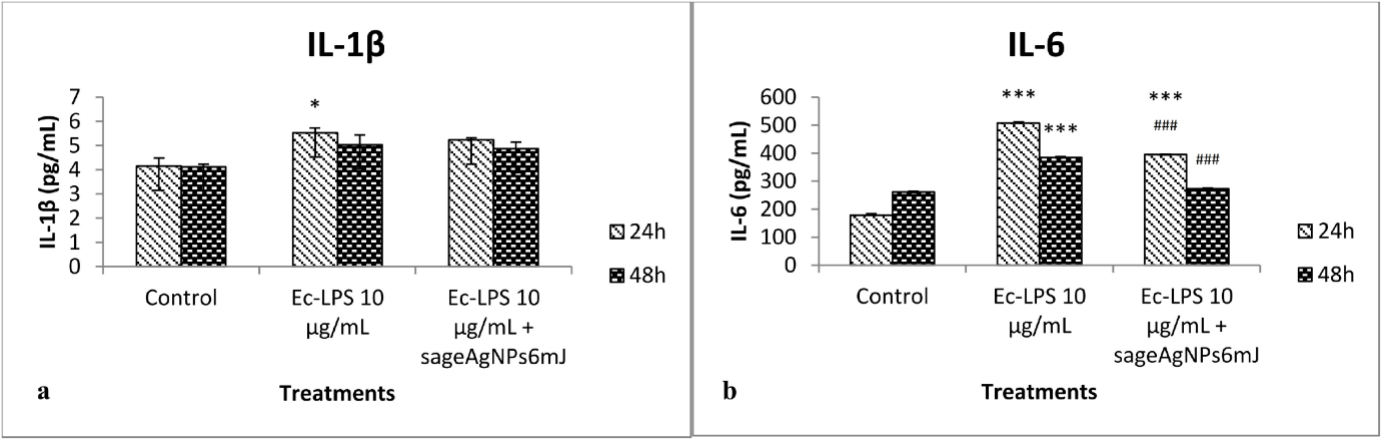
IL-1β (a) and IL-6 values (b) in cell culture supernatants after treatment with EcLPS (10 µg·mL^−1^) and EcLPS + 1 × MIC _Sage_AgNPs_6mJ_ for 24 and 48 h, expressed as pg·mL^−1^. ^*^p < 0.05, ^***^p < 0.001, treatment vs control; ^###^*p* < 0.001, _Sage_AgNPs_6mJ_+EcLPS vs EcLPS.

Proinflammatory cytokine measurements (IL-1β and IL-6) were performed in the cell cultures treated with EcLPS and _Sage_AgNPs_6mJ_. _Sage_AgNPs_6mJ_ treatment discreetly reduced IL-1β levels compared to EcLPS treatment after both exposure times (Figure 5a). However, the levels of IL-6 were significantly reduced after _Sage_AgNPs_6mJ_ treatment compared to EcLPS treatment (Figure 5b). Upon 48 h of treatment, the IL-6 levels were reduced to the levels of untreated control, indicating a stronger anti-inflammatory impact after longer treatment duration.

## Discussion

This study describes the synthesis of AgNPs via PLAL using aqueous sage extract as a stabilizing medium, thereby integrating a contaminant-free physical approach with bioactive plant-derived compounds. This strategy enables the potential preservation and enhancement of both nanoparticle and phytochemical properties, while avoiding chemical reagents. Furthermore, the work provides a novel, comprehensive evaluation of these nanoparticles in the context of periodontal therapy by simultaneously assessing their antibacterial, anti-inflammatory, and regenerative effects on primary human gingival fibroblasts in vitro*.*

Our previous comprehensive characterization of aqueous sage extract obtained by lyophilization revealed high levels of total phenolics (162.00 ± 3.51 mg GAE·g^−1^ DE) and flavonoids (39.47 ± 4.31 mg QE·g^−1^ DE), confirming that it can be suitable for green nanoparticle synthesis [13]. The HPLC quantification of selected phenolic acids identified high concentrations of rosmarinic and caffeic acids, while vanillic and ferulic acids were below the detection limit, probably due to the lower polarity of the used solvent. We chose water extraction over organic solvents to reach a balance between safety and bioactivity; the therapeutic potential of the obtained nanoparticles could be compromised by the organic solvents, even though they usually extract higher levels of phytoconstituents [30]. In contrast, aqueous extraction relies on eco-friendly principles that reduce toxicity and support the biocompatibility of the obtained nanoparticles. The high concentration of rosmarinic acid in our extract is particularly important for AgNP synthesis and stabilization since it possesses potent reducing properties to convert Ag^+^ ions to AgNPs [25]. This reduction is enhanced by caffeic acid, which acts synergistically to facilitate nanoparticle formation and stabilization [31–32], suggesting that the sage extract obtained in our study could increase stability and bioactivity of silver nanoparticles. Additionally, our previous study revealed a relatively high content of camphor (approximately 15%), suggesting that, besides phenolics, terpenoids may also contribute to nanoparticle synthesis [13]. To the best of our knowledge, there are no studies that directly investigated the role of camphor in the synthesis of AgNPs. However, literature findings indicate that plant extracts rich in camphor may contribute to nanoparticle formation by acting as both reducing and stabilizing agent, suggesting its role in AgNP synthesis. Other terpenes and flavonoids were present in low concentrations and were therefore considered unlikely to contribute to the AgNP synthesis [13,33].

To assess the influence of sage extract and laser energy on the AgNP synthesis efficiency, four samples of AgNPs were synthesized. Higher laser pulse energies and sage liquid environment synergistically increased nanoparticle concentration while reducing particle size. The simultaneous reduction in particle size with higher laser energy is in line with our previous results for citrate-stabilized AgNPs [14], highlighting the influence of laser operating parameters on size and concentration of the nanoparticles. A shorter pulse time, 150 ps pulses in our experimental setup, leads to quick evaporation, minimum heating of the target area, and very low absorbed energy, thereby enhancing ablation efficiency. The aqueous liquid sage extract environment significantly influenced both concentration and size parameters. It also improved their dispersity and stability, as shown by DLS and TEM, confirming that sage acts as a reducing and stabilizing agent during the synthesis. The differences in the measured dH and TEM diameters reside in the fact that the resulting dH value represents the nanoparticle size in a hydrated state, along with the adjacent layers of water or sage extract molecules, expanding the hydrodynamic diameter, which cannot be observed by TEM. These differences are more pronounced for _dw_AgNPs_6mJ_, suggesting a certain extent of particle agglomeration in suspension, which was confirmed by TEM imaging. Stronger agglomeration of _dw_AgNPs_6mJ_ one month after synthesis compared to _Sage_AgNPs_6mJ_ confirmed that sage acts as a potent reducing and stabilizing agent during the synthesis. As mentioned above, this phenomenon is probably a result of high contents of rosmarinic and caffeic acids in the sage extract since they act as efficient capping agents that stabilize nascent AgNPs during the critical post-ablation period [25,31–32]. AgNPs obtained by PLAL in liquid sage extract environment, with smaller particle size and higher concentration, could possesses several biological benefits. While the higher concentration provides greater local therapeutic doses, the smaller particle size allows for greater cellular internalization by endocytosis [34]. The narrow size distribution of _Sage_AgNPs_6mJ_ could also contribute to the consistent biological outcomes.

Since microbial dysbiosis is the primary step in periodontal disease pathogenesis, we first examined the potential of the obtained nanoparticles to inhibit the growth of common periodontal pathogens. It was reported that the antibacterial activity of AgNPs is directly proportional to their concentration and inversely proportional to their size, which is consistent with our findings [35–36]. AgNPs obtained with the lower laser energy had lower concentrations and larger diameters, and did not display antibacterial activities even at the highest tested concentrations. The antibacterial efficacy of AgNPs depends on their concentration but also on their size. Smaller nanoparticles exhibit a higher surface-area-to-volume ratio, which enhances their reactivity and interaction with bacterial cells. Moreover, reduced particle size enables more efficient penetration of the bacterial cell membrane, thereby contributing to their antimicrobial activity [37–38]. In contrast, both AgNP samples obtained with higher laser energy had higher concentrations and smaller particle sizes, and showed high inhibitory potential; _Sage_AgNPs_6mJ_ exhibited the highest antibacterial effects among all tested nanoparticles. The superior antibacterial efficacy of _Sage_AgNPs_6mJ_ against the tested bacterial strains can be attributed to several mechanisms. Sage’s phenolic compounds, especially rosmarinic and caffeic acids, increase the antimicrobial activity through bacterial cell membrane damage and interaction with bacterial metabolic processes [39–40]. These compounds contribute to the antimicrobial mechanisms of AgNPs, including disruption of bacterial cell wall synthesis, interference with DNA replication, and generation of reactive oxygen species (ROS) [36]. The same MIC and MBC values observed for _Sage_AgNPs_6mJ_ indicate bactericidal activity rather than bacteriostatic effects. This characteristic is valuable for the treatment of periodontal disease as it ensures complete bacterial elimination rather than growth inhibition, thereby reducing the risk of bacterial resistance development [41]. Remarkably, _Sage_AgNPs_6mJ_ demonstrated a more potent antibacterial activity against *F. nucleatum* and *A. actinomycetemcomitans* in comparison to the positive control, 0.2% chlorhexidine, outlining their efficacy against periodontal pathogens.

Despite the admirable antibacterial properties of AgNPs synthesized with the higher laser energy of 6 mJ, their use in dental medicine could be limited due to bystander toxicity [42]. The results of cell viability testing showed that only _Sage_AgNPs_6mJ_ did not reduce cell viability after both treatment times at 1 × MIC concentration, suggesting that the sage extract used for AgNP synthesis mitigates their cytotoxicity. This result is probably the effect of sage on the physicochemical properties of the AgNPs, as well as the effect of sage constituents on the cell viability (Figure 2a). However, a higher concentration of _Sage_AgNPs_6mJ_ also significantly reduced the percentage of viable cells, outlining the concentration limit. Previous studies also showed that the toxicity of AgNPs strongly depends on the size, concentration, and surface modification, and that the synthesis with phytochemicals could reduce their toxicity and improve biocompatibility [43–44]. _Sage_AgNPs_6mJ_ exhibited significantly lower cytotoxic effects than their uncoated counterparts, probably by attenuating the release of Ag^+^ ions and through sage’s inherent antioxidant activity that helps scavenge ROS, thus decreasing oxidative cellular damage [45–46]. In biological media, silver speciation is highly dynamic due to interactions with proteins and ions, which makes direct quantification of free Ag^+^ ions challenging [47].

Interestingly, _Sage_AgNPs_6mJ_ demonstrated toxicity toward bacterial cells without decreasing the viability of GFCs at the same concentration, thus achieving antimicrobial efficacy without cellular damage. Selective toxicity toward periodontal Gram-negative pathogens could originate from both structural and biochemical differences between prokaryotic and eukaryotic cells. Their negatively charged envelope enables electrostatic interaction with AgNPs, promoting membrane destabilization and their intake; internalized AgNPs induce high levels of ROS, damaging essential biomolecules [48]. In contrast, eukaryotic cells internalize nanoparticles primarily via endocytosis, enabling partial localization in endo-lysosomal compartments, which, together with more efficient repair mechanisms, contribute to the selective antibacterial activity of AgNPs with comparatively lower cytotoxicity toward mammalian cells [49]. This selectivity is essential for potential applications where treatment should eliminate pathogenic bacteria and maintain the viability of gingival cells responsible for tissue repair and regeneration. The differences between bacterial and eukaryotic cell metabolisms may be the cause of this selectivity. Eukaryotic cells have strong antioxidant systems that can neutralize ROS as long as particle concentrations stay within therapeutic ranges [50]. The sage extract compounds enhance this selectivity by providing additional antioxidant protection to human cells [13] while maintaining antimicrobial efficacy against bacteria.

Since only _Sage_AgNPs_6mJ_ displayed selective toxicity towards tested bacteria and GFCs, the subsequent biological evaluations regarding proliferation, collagen production, and inflammation reduction, were performed exclusively on _Sage_AgNPs_6mJ_. The cytotoxic effects observed with AgNPs synthesized in deionized water would hamper interpretation of these biological parameters since cellular damage would challenge their assessment. The increased proliferation of GFCs, demonstrated by Ki67 staining after 24 and 48 h of treatment indicated that _Sage_AgNPs_6mJ_ at 1 × MIC concentration promote cell division without inducing cytotoxic effects. The concentration- and size-dependent influence of AgNPs on the proliferation of human peripheral blood lymphocytes, human keratinocytes, and dermal fibroblasts was previously reported [14,42,51–52]; it was mostly attributed to the suppression of proinflammatory pathways, the activation of anti-inflammatory pathways, and the selective inhibition of the cyclooxygenase-2 (COX-2) pathway [53]. Sage extract used for the synthesis likely contributes to this effect by increasing the cyclin D1 expression level, thus accelerating and promoting cellular proliferation [54]. Additionally, rosmarinic acid promotes cell growth and proliferation by lowering inflammation, preserving mitochondrial function, and triggering the PI3K/Akt signaling pathway [55–56]. Increased Ki67 expression after both 24 and 48 h of treatment suggests sustained proliferative activity, indicating that _Sage_AgNPs_6mJ_ maintain their bioactivity over extended treatment periods. This effect is crucial for periodontal regeneration as it allows for continuous tissue repair and remodeling of the extracellular matrix (ECM). However, consistent with cell viability results, higher concentration of 2 × MIC decreased cell proliferation, probably by excessive ROS production, which overcomes cellular defense mechanisms, outlining the concentration limit.

Collagen production and remodeling are crucial stages in tissue restoration and regeneration [57]. Besides the reported role of AgNPs in improved collagen deposition during wound healing [58–59], using plant extracts during nanoparticles synthesis was found to promote wound healing and collagen modeling in vivo [60]; but, to the best of our knowledge, the effects of AgNPs synthesized in the presence of sage on collagen production have not been previously reported. In the present study, enhanced intracellular total collagen production and ECM deposition of type-I collagen following both treatment times with _Sage_AgNPs_6mJ_ reflect important advancements for periodontal regeneration. Previous studies also reported positive effects of AgNPs on collagen synthesis and deposition in vivo by activation of the TGF-β/Smad signaling pathway, a key regulator of collagen gene expression [61–62]. Furthermore, sage extract enhances the bioactivity of AgNPs by stimulating fibroblast proliferation and collagen synthesis [54], thus contributing to the pro-collagen effect.

During the progression of periodontal disease, the increase in secretion of the pro-inflammatory cytokines IL-1β and IL-6 contributes to periodontal detachment and alveolar bone loss, recognizing them as targets for periodontal disease treatment [63–65]. In the present study, modest reduction in IL-1β and significant reduction in IL-6 levels were detected after 24 h of treatment, while a longer treatment time of 48 h reduced the levels IL-6 to the those of the untreated control. The anti-inflammatory effect of _Sage_AgNPs_6mJ_ observed in our study can be attributed to the AgNPs’ capacity to interact with the NF-κB signaling pathway by preventing its nuclear translocation and activation [66]. Since NF-κB upregulates the expression of pro-inflammatory genes, including IL-1β and IL-6, the inhibition of its nuclear translocation could suppress their transcription, leading to inflammation reduction [67]. Additionally, rosmarinic acid exhibits potent anti-inflammatory properties by downregulation of the NF-κB signaling pathway and the inhibition of COX-2 activity, thus contributing to the anti-inflammatory effects of the silver nanoparticles [68]. It was previously demonstrated that high levels of IL-6 upregulate the expression of certain matrix metalloproteinases, enzymes involved in the degradation of the extracellular matrix, causing collagen degradation and disturbed tissue remodeling [69–70]. The combined effects of _Sage_AgNPs_6mJ_ on interleukin level reduction and increased collagen synthesis and ECM deposition, especially after the longer treatment time, show a promising restorative potential.

While the findings of the present work highlight the promising biological potential of sage-synthesized AgNPs by PLAL, several aspects should be taken into account when interpreting the results. All biological evaluations were performed in vitro, which cannot entirely imitate the complex microenvironment of periodontal tissues in vivo. In addition, the biological analyses were focused primarily on the AgNPs synthesized with the higher laser energy (_Sage_AgNPs_6mJ_) since nanoparticles obtained in deionized water exhibited cytotoxic effects that could hinder the interpretation of cellular responses. While this approach enabled a clearer evaluation of regenerative and anti-inflammatory effects, it limited direct biological comparisons among all synthesized nanoparticle formulations. Moreover, under clinical conditions, maintaining a precise intrapocket concentration under continuous gingival crevicular fluid flow is a key challenge. However, this can be addressed by incorporating nanoparticles into controlled-release local delivery systems, enabling sustained therapeutic levels while avoiding cytotoxic peaks. Future studies incorporating optimized concentrations or surface modifications may allow for a broader comparative biological assessment.

## Conclusion

AgNPs synthesized by picosecond PLAL in aqueous *Salvia officinalis* extract with laser pulse energies of 6 mJ exhibited superior properties compared to those produced in water, including reduced particle size, higher yield, and enhanced antibacterial activity against key periodontal pathogens. Notably, at MIC concentration, these nanoparticles simultaneously promoted gingival fibroblast proliferation, collagen synthesis, and anti-inflammatory responses, indicating a desirable therapeutic profile. To the best of our knowledge, this is the first report of PLAL sage-mediated AgNP synthesis, demonstrating a novel, contaminant-free strategy that integrates nanotechnology with plant-derived phytochemicals to achieve positive therapeutic effects relevant for periodontal therapy. Future work should be focused on in vivo validation, mechanistic insights, and the development of clinically translatable formulations for targeted periodontal applications.

## Data Availability

Data generated and analyzed during this study is available from the corresponding author upon reasonable request.
